# Combining nonsense mutation suppression therapy with nonsense-mediated decay inhibition in neurofibromatosis type 1

**DOI:** 10.1016/j.omtn.2023.06.018

**Published:** 2023-06-26

**Authors:** Sara H. Osum, Eunice I. Oribamise, Stanislas M.A.S. Corbière, Mandy Taisto, Tyler Jubenville, Alex Coutts, Mark N. Kirstein, James Fisher, Christopher Moertel, Ming Du, David Bedwell, David A. Largaespada, Adrienne L. Watson

**Affiliations:** 1 Masonic Cancer Center, Department of Pediatrics, University of Minnesota, 2-191 Moos Tower, 515 Delaware Street SE, Minneapolis, MN 55455, USA; 2 Recombinetics Inc., 3388 Mike Collins Drive, #1, Eagan, MN 55121, USA; 3 Department of Experimental and Clinical Pharmacology, College of Pharmacy, University of Minnesota, Room 459, 717 Delaware Street SE, Minneapolis, MN 55414, USA; 4 Department of Biochemistry and Molecular Genetics, University of Alabama at Birmingham, Bevill Biomedical Research Building Room 432A, 845 19^th^ Street South, Birmingham, AL 35294, USA

**Keywords:** MT: Delivery Strategies, neurofibromatosis, nonsense suppression, minipig, preclinical models, pharmacokinetics, nonsense mutations

## Abstract

Neurofibromatosis type 1 (NF1) results from germline mutations in the tumor-suppressor gene *NF1* and predisposes patients to developing nervous system tumors. Twenty percent of NF1 patients harbor nonsense mutations resulting in premature termination codons (PTCs). Nonsense suppression therapies can facilitate ribosomal readthrough of PTCs to restore full-length protein, but their potential in NF1 is underexplored. We developed a minipig model of NF1 carrying a PTC to test whether nonsense suppression could restore expression of the *NF1*-encoded protein neurofibromin *in vitro* and *in vivo*. Nonsense suppression did not reliably increase neurofibromin in primary *NF1*^*−/−*^ Schwann cells isolated from minipig neurofibromas but could reduce phosphorylated ERK. Gentamicin *in vivo* produced a similar plasma pharmacokinetic profile to humans and was detectable in clinically relevant tissues, including cerebral cortex, sciatic nerve, optic nerve, and skin. In gentamicin-treated animals, increased neurofibromin expression was seen in the optic nerve. Nonsense-mediated decay (NMD) causes degradation of transcripts with PTCs, which could impede nonsense suppression therapies. Nonsense suppression in combination with NMD inhibition restored neurofibromin protein expression in primary *NF1*^*−/−*^ Schwann cells isolated from minipig neurofibromas. Thus, the effectiveness of nonsense suppression therapies can be improved in NF1 by the concurrent use of NMD inhibitors.

## Graphical abstract

**Figure:**
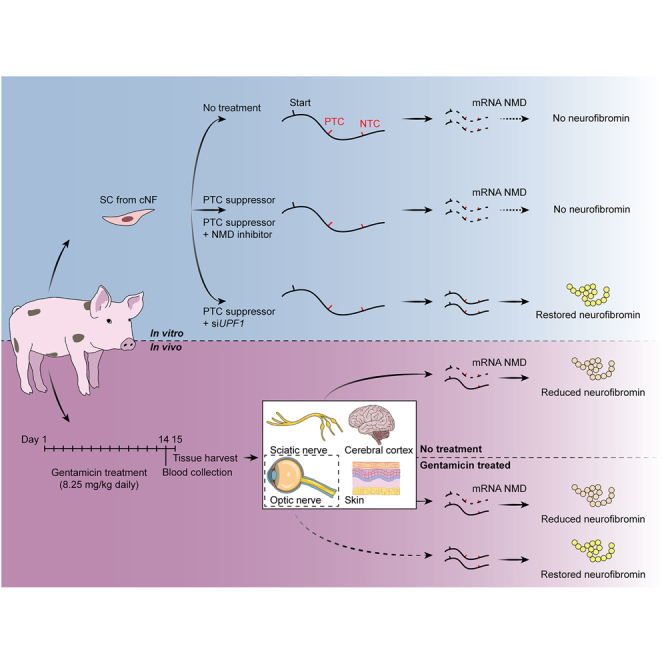

## Teaser

Watson and colleagues show nonsense mutation suppression has the potential to treat the underlying cause of neurofibromatosis type 1 (NF1) in patients with *NF1* nonsense mutations. In a minipig model, combination suppression of nonsense mutations and nonsense-mediated decay increases translation of neurofibromin and has potential to treat NF1 patients.

## Introduction

Over 20% of patients with neurofibromatosis type 1 syndrome (NF1) have a nonsense mutation in the *NF1* gene.^1^ Nonsense mutations result in premature termination codons (PTCs) and the production of truncated proteins or degradation of the RNA transcript by nonsense-mediated decay (NMD).^2^ One approach to treat genetic diseases associated with nonsense mutations in tumor-suppressor genes like *NF1* is through pharmacologic nonsense suppression, which induces the translational machinery to recode the PTC as a sense codon and allow production of full-length protein.^3^ Many drugs have been shown to facilitate nonsense suppression, including aminoglycoside antibiotics, which bind to the eukaryotic ribosome with a lower affinity than prokaryotes, thus allowing translation with some error at PTCs.^3^ Other compounds, like ataluren, have been developed specifically for enhancing readthrough of PTCs, and to avoid toxicity issues that accompany long-term aminoglycoside treatment.^4^ To date, the focus of research and clinical trials with these drugs has been to treat cystic fibrosis (CF) and Duchenne muscular dystrophy (DMD).^5^^,^^6^^,^^7^^,^^8^ However, the therapeutic application of these drugs in NF1 patients with nonsense mutations has yet to be explored.

We recently developed an NF1 Ossabaw minipig with a genetically engineered loss of function mutation in the *NF1* gene that exhibits clinical features of human NF1. This includes cutaneous neurofibromas containing Schwann cells that have undergone loss of heterozygosity (LOH) of the wild-type (WT) *NF1* allele and show complete loss of the *NF1* gene product, neurofibromin.^9^ In previous studies, our model has been shown to be useful for evaluating pharmacokinetics and pharmacodynamics of MEK inhibition for NF1 patients in clinically relevant tissues.^10^ For preclinical testing of nonsense suppression therapies in NF1, we developed a new swine model of NF1 carrying an R1947X nonsense mutation (*NF1*^*NS/+*^) found in 62 or 8,100 unrelated and symptomatic NF1 patients and evaluated a panel of nonsense suppression therapies for their ability to restore neurofibromin protein expression *in vitro* and *in vivo*.^1^^,^^11^^,^^12^^,^^13^^,^^14^^,^^15^

In this study, we evaluated the efficacy of nonsense suppression therapy in the NF1 minipig model using the readthrough compounds gentamicin, G418, and ataluren, in primary heterozygous Schwann cells derived from normal sciatic nerve and primary Schwann cells with LOH (*NF1*^*NS/−*^) derived from cutaneous neurofibromas. Results were correlated with *in vivo* testing of gentamicin to determine the pharmacodynamic effects in clinically relevant tissues including optic nerve, sciatic nerve, cerebral cortex, and skin.

A barrier to the effective application of nonsense suppression therapies is degradation of PTC-containing transcripts by NMD. Pharmacologic inhibition of NMD has been shown to augment nonsense suppression and improve expression of mutant transcripts, eliciting a functional effect in preclinical models of DMD and CF.^7^^,^^16^^,^^17^ To test this in NF1, we investigated the efficacy of combining nonsense suppression with NMD inhibition and found that this restored neurofibromin protein expression in primary *NF1*^*NS/−*^ Schwann cells isolated from minipig neurofibromas. NMD was achieved genetically using small interfering RNA (siRNA) targeting *UPF1* (si*UPF1)* and pharmacologically with NMD inhibitors (NMDI)-1, VG1, and NMDI14. However, full-length neurofibromin expression was only achieved with si*UPF1* in combination with G418.

This is the first report of restoring full-length neurofibromin protein expression using nonsense suppression. These results demonstrate the potential of nonsense suppression therapy to both eradicate the underlying genetic cause of NF1 and cure existing manifestations of the disease in NF1 patients and highlights the need for improved pharmacologic agents for nonsense suppression and NMD inhibition.

## Results

**Figure 1:**
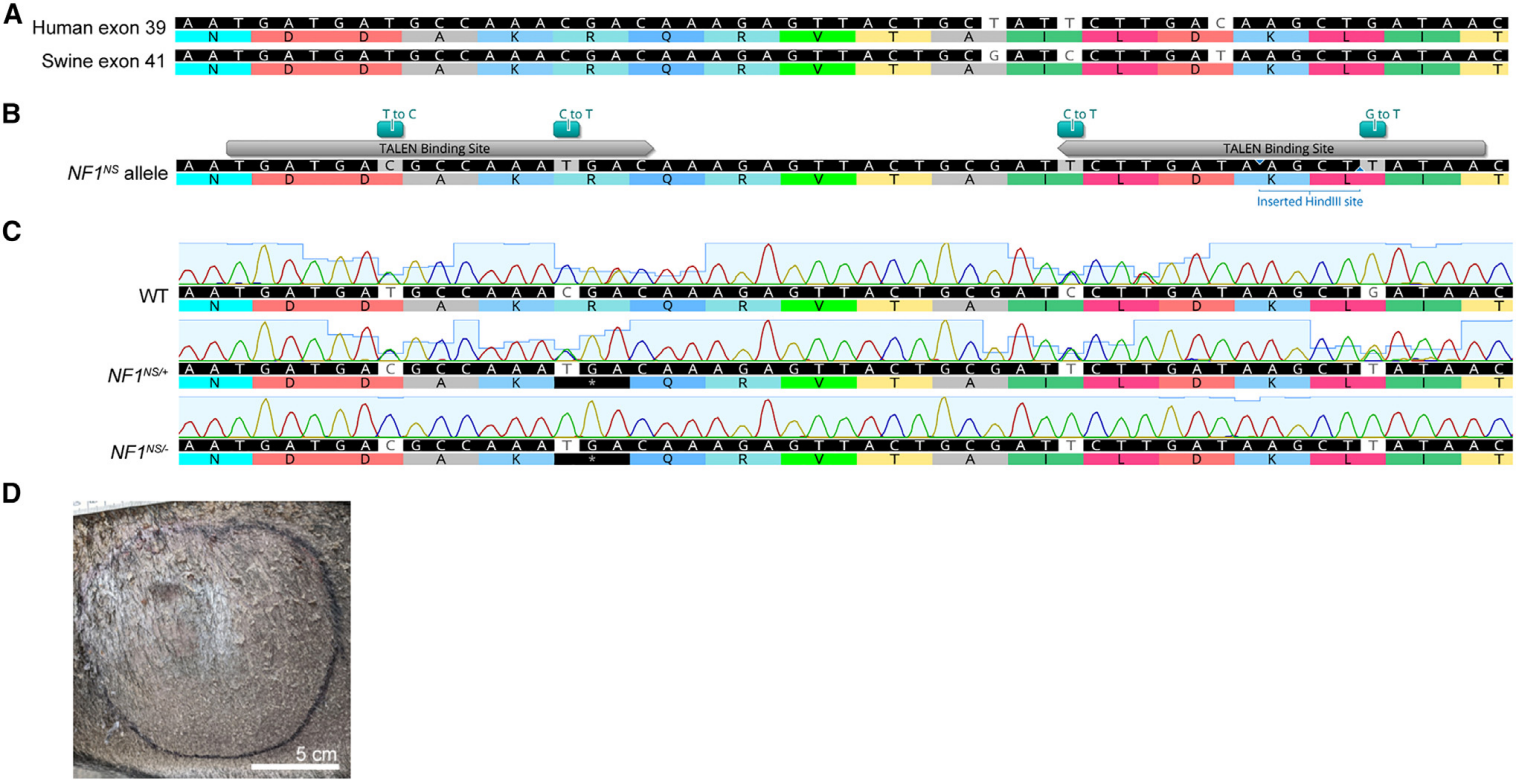
Development of *NF1*^*NS/+*^ (NF1) minipigs (A) Human exon 39 and swine exon 41 of the NF1 gene show 100% amino acid homology. Partial exon regions are represented; non-highlighted gray letters, differences in nucleotide sequences. (B) *NF1*^*R1947∗*^ (*NF1*^*NS*^) gene edited allele after homologous recombination (HR) using HDR oligonucleotide containing silent mutations and R1947X mutation. Non-highlighted gray letters, differences in nucleotide sequences compared with wild-type (WT) swine exon; superior blue box, changes induced by HR; blue square bracket and arrowhead, RFLP site; black ∗ box, stop codon; gray arrows, TALEN binding sites. (C) Sanger sequencing analysis of WT, *NF1*^*R1947∗/+*^ (*NF1*^*NS/+*^), *NF1*^*R1947∗/−*^ (*NF1*^*NS/−*^) Schwann cells from F1 minipigs. Partial exon regions are represented; non-highlighted gray letters, differences in nucleotides between animals. (D) Gross image of cutaneous neurofibroma in NF1 minipig. Black marker denotes outline of the tumor.

**Figure 2:**
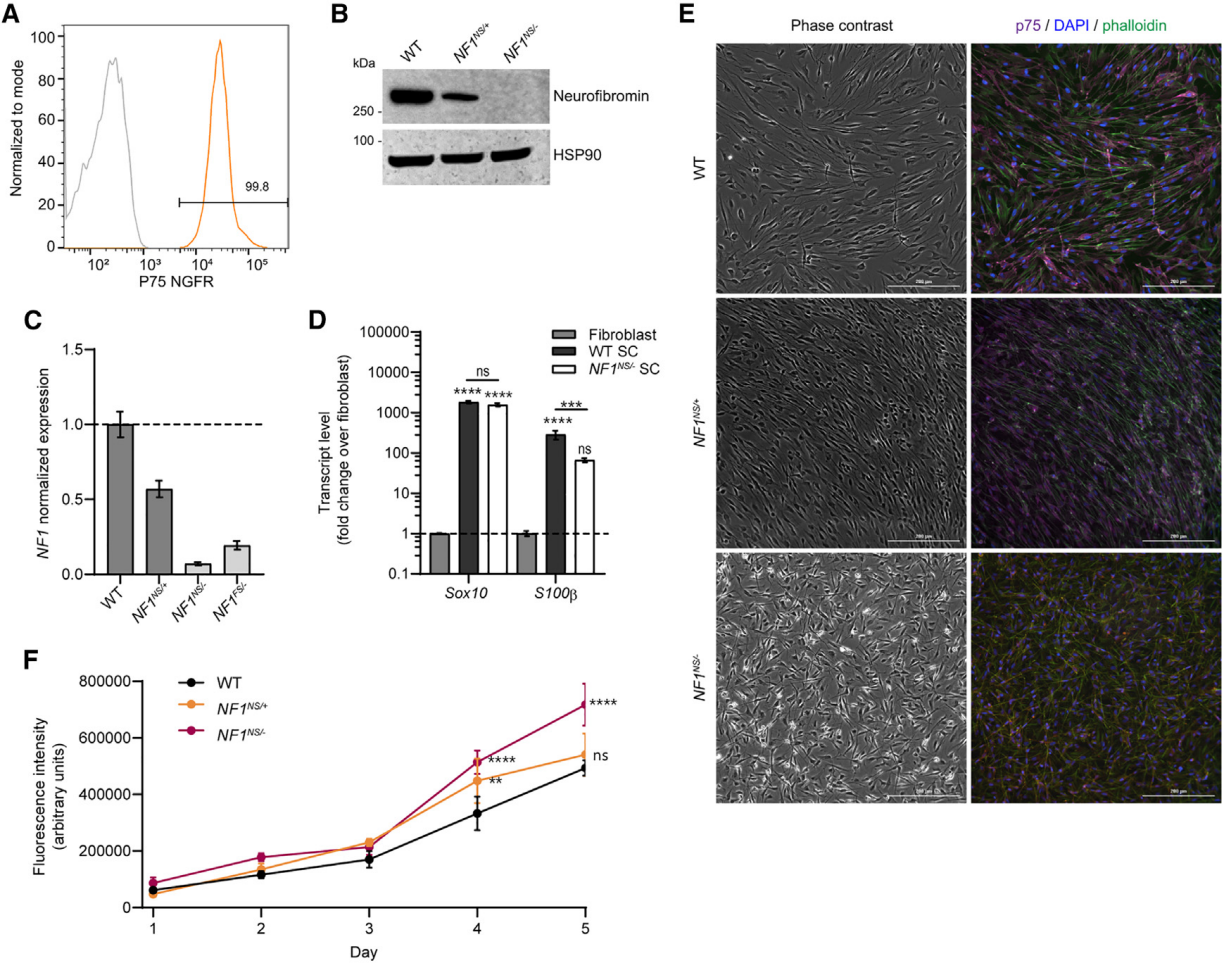
Characterization of primary Schwann cells isolated from NF1 minipigs (A) Representative flow cytometry plot of p75 NGFR expression in primary Schwann cells after magnetic selection compared with isotype control. (B) Representative western blot showing expression of neurofibromin protein in primary Schwann cell cultures from WT or *NF1*^*NS/+*^ sciatic nerve, or cutaneous neurofibroma (*NF1*^*NS/−*^ SC). HSP90, heat shock protein 90. (C) RT-qPCR analysis of *NF1* gene expression in Schwann cells from WT and *NF1*^*NS/+*^ minipigs, and cNF Schwann cells with LOH from *NF1*^*NS/+*^ or *NF1*^*FS/+*^ minipigs. Error bars represent mean and SD from three technical replicates. (D) RT-qPCR analysis of canonical SC markers *S100b* and *Sox10* in WT Schwann cells and cNF Schwann cells with LOH from *NF1*^*NS/+*^ minipigs relative to WT dermal fibroblasts. Error bars represent mean and SD from three technical replicates. Two-way ANOVA with Dunnett’s multiple comparison test (ns, not significant with p value >0.05; ∗p value ≤ 0.05; ∗∗p value ≤ 0.01; ∗∗∗p value ≤ 0.001; ∗∗∗∗p value < 0.0001). (E) Representative brightfield and immunofluorescence images of phalloidin and p75 NGFR staining showing purity of Schwann cells and abnormal morphology and orientation of Schwann cells in *NF1*^*NS/−*^ Schwann cells compared with WT and *NF1*^*NS/+*^ Schwann cells. (F) Proliferation assay showing increased proliferation in *NF1*^*NS/−*^ Schwann cells compared with WT and *NF1*^*NS/+*^ Schwann cells. Error bars represent SD of three technical replicates. Two-way ANOVA with Dunnett’s multiple comparison test, with individual variances computed for each comparison (ns, not significant with p value >0.05; ∗p value ≤ 0.05; ∗∗p value ≤ 0.01; ∗∗∗p value ≤ 0.001; ∗∗∗∗p value < 0.0001).

To develop a swine model of NF1 with a nonsense mutation suitable for the evaluation of nonsense mutation suppression therapies, we utilized transcription activator-like effector nucleases (TALENs) to create a double-stranded break and a 90-nucleotide homology-directed repair template to introduce the *NF1*^*R1947X*^ nonsense mutation (*NF1*^*NS*^*)* into fetal Ossabaw minipig fibroblasts, resulting in an in-frame UGA codon (Figures 1A and 1B). Four silent mutations were also engineered to prevent TALEN rebinding and nuclease cutting. Heterozygous clones were identified, and somatic cell nuclear transfer was used to generate clonal *NF1*^*NS/+*^ (NF1) offspring (Figure 1C). NF1 minipigs were viable, fertile, and transmitted the mutant allele to future generations with Mendelian frequencies. The phenotype of these NF1 minipigs was very similar to what was previously reported, with 100% of the animals displaying café au lait macules (CALMs) and a subset of NF1 minipigs developed lesions resembling NF1-associated cutaneous neurofibromas (Figure 1D).^9^ Primary Schwann cells were isolated and cultured from WT and NF1 sciatic nerve, as well as cutaneous neurofibromas (cNFs).^9^ Schwann cells were purified from contaminating cells using p75 nerve growth factor receptor (NGFR) antibody-mediated magnetic cell separation and confirmed to be >95% pure by flow cytometry (Figure 2A).^9^^,^^18^ A subset of Schwann cells isolated from cutaneous neurofibromas showed loss of the WT allele by DNA sequencing (Figure 1C) and subsequent loss of neurofibromin protein expression (Figure 2B). Based on the loss of surrounding single nucleotide polymorphisms (SNPs) at this locus, this loss was either by allelic conversion or a large deletion. There was no evidence of truncated neurofibromin protein product in Schwann cells with LOH from NF1 minipigs with a nonsense (*NF1*^*NS/−*^) or frameshift (*NF1*^*FS/−*^) mutation, as shown by western blotting with an antibody that recognizes the N terminus of the neurofibromin protein (Figure S1). Transcript levels of *NF1* were decreased by roughly 50% in *NF1*^*NS/+*^ Schwann cells from NF1 minipig sciatic nerve compared with WT, and by 90% in *NF1*^*NS/−*^ and *NF1*^*FS/−*^ Schwann cells (Figures 2C and S2A). Transcript levels of canonical Schwann cell markers, including *Sox10*, *S100ß,* and *NGFR,* were expressed highly in Schwann cell cultures compared with fibroblast cultures, further confirming their identity (Figures 2D and S2C–S2E). Interestingly, *S100ß* expression was significantly decreased in *NF1*^*NS/−*^ by RT-qPCR, but RNA sequencing analysis showed no significant difference in expression of this transcript among WT, *NF1*^*NS/+*^*, NF1*^*NS/−*^*,* and *NF1*^*FS/−*^ Schwann cells (Figure S2B)*.* Schwann cells derived from cutaneous neurofibromas showed altered morphology, disorganized architecture, and increased proliferation compared with WT and *NF1*^*NS/+*^ sciatic nerve-derived Schwann cells (Figures 2E and 2F). Five out of six primary Schwann cell cultures derived from neurofibromas displayed altered morphology and disorganized architecture without evidence of LOH or loss of neurofibromin protein expression (Table S1). All further work was performed with Schwann cells that showed LOH by DNA sequencing and complete loss of neurofibromin protein expression by western blot.

To determine whether nonsense suppression therapy could restore full-length neurofibromin protein and reduce downstream MAPK signaling in NF1 minipigs, we tested several nonsense suppression therapies *in vitro*: ataluren, gentamicin, and G418. These drugs were chosen because there are significant data on their use *in vitro*, *in vivo,* and in clinical trials.^3^^,^^4^^,^^5^^,^^6^^,^^17^^,^^19^^,^^20^^,^^21^ *NF1*^*NS/+*^ Schwann cells from sciatic nerve and *NF1*^*NS/−*^ Schwann cells from cutaneous neurofibromas were treated with ataluren, gentamicin, or G418 at multiple concentrations, as indicated, for a time course of 24, 48, or 72 h (Table S2). While certain *NF1*^*NS/+*^ Schwann cell cultures from sciatic nerve appeared to show increased neurofibromin with gentamicin or G418 at certain doses and/or time points, the results were not consistent between replicates or did not exhibit a dose- or time-dependent increase (Figures S3A–S3I). For example, neurofibromin increased in all three replicates treated with 100 μg/mL G418 at 48 h, but not with 200 μg/mL and not at 72 h (Figures S3A–S3C). Moreover, p-ERK paradoxically increased at this time point and concentration (Figures S3A–S3C). Gentamicin treatment resulted in a linear increase in neurofibromin at 48 h in one replicate (Figure S3F), but this result was not replicated in other *NF1*^*NS/+*^ Schwann cell cultures (Figures S3D and S3E). We next performed these experiments using *NF1*^*NS/−*^ Schwann cells from cutaneous neurofibromas that showed LOH, as it is easier to visualize restoration of neurofibromin expression when baseline expression is zero. We demonstrated that even in this case, there was no indication of nonsense suppression through expression of neurofibromin, and there was not a corresponding reduction in p-ERK (Figure S4).

**Figure 3:**
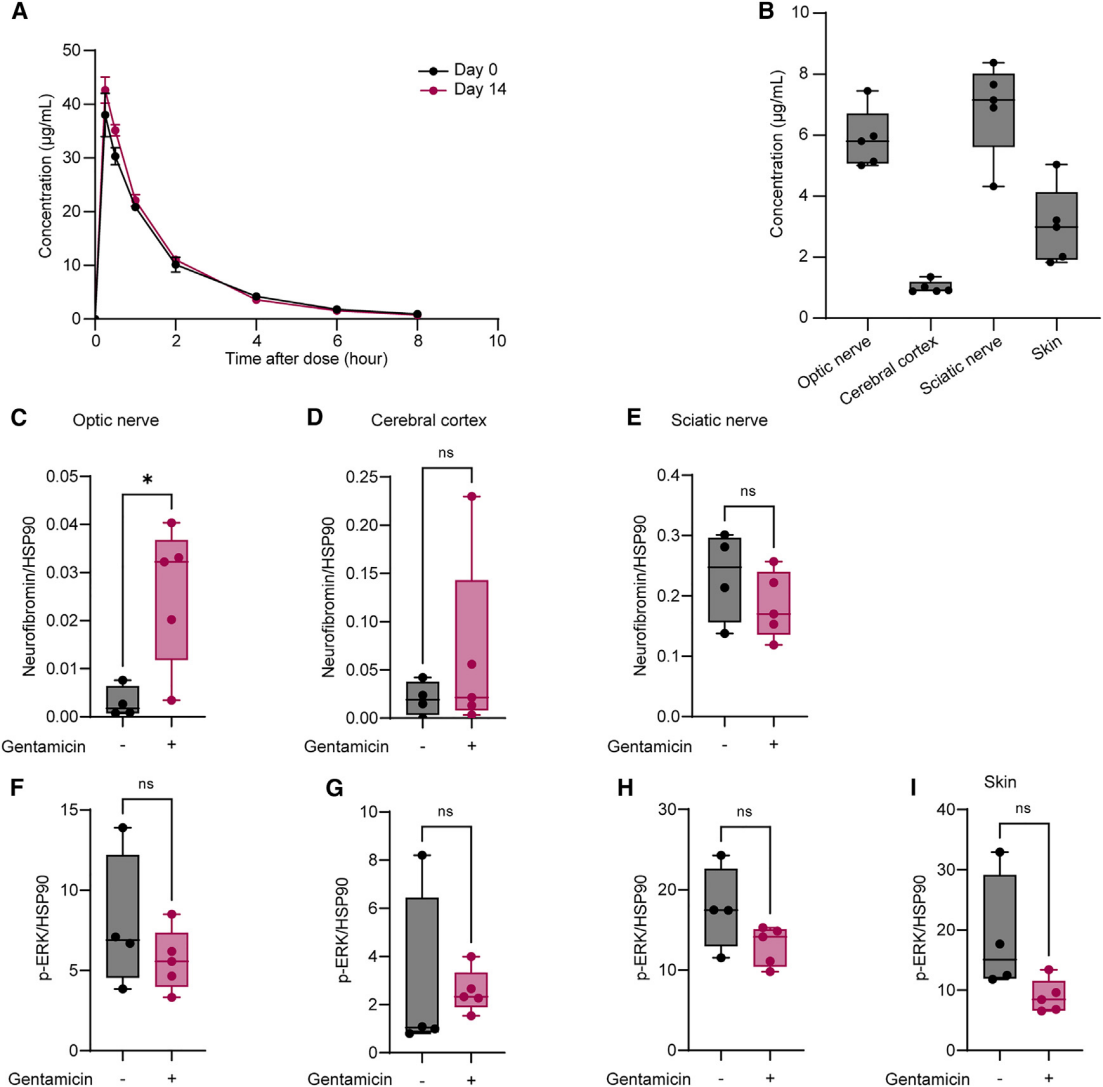
Gentamicin increases neurofibromin expression in optic nerve tissue from NF1 minipigs (A) Mean plasma concentration-time curves on day 1 and day 14 after daily subcutaneous administration of gentamicin. Error bars represent SEM from five biological replicates. (B) Gentamicin concentration in NF1 minipig tissues after 14 days. Samples were collected 30 min after gentamicin administration. Error bars represent SEM from five biological replicates. Western blot quantification of neurofibromin (C)–(E) and p-ERK (F)–(I) protein expression in optic nerve (C) and (F), cortex (D) and (G), sciatic nerve (E) and (H), and skin (I) from gentamicin-treated (n = 5) and untreated control (n = 4) animals, normalized to HSP90. Neurofibromin was not detected in skin samples. Error bars represent mean and SD. Unpaired t-test with Welch’s correction was performed (ns, not significant with p value > 0.05; ∗p value ≤ 0.05; ∗∗p value ≤ 0.01; ∗∗∗p value ≤ 0.001; ∗∗∗∗p value < 0.0001).

We next performed a pharmacokinetic (PK) and pharmacodynamic (PD) analysis of gentamicin to determine whether we could achieve a PD effect of nonsense suppression in NF1 minipig tissues based on a pre-determined target plasma concentration. Gentamicin has shown the ability to suppress PTCs in other preclinical models and in clinical trials.^3^ In murine models of DMD and CF, subcutaneous administration of gentamicin once daily was shown to suppress PTCs at a dose of 34 mg/kg and peak plasma concentration (C_max_) of 40–60 μg/mL.^22^^,^^23^ In human patients with DMD or CF, once daily intravenous infusion of gentamicin at 7.5–10 mg/kg resulted in a C_max_ of 20–40 μg/mL.^5^^,^^6^^,^^24^ To determine a dose of gentamicin that reaches a C_max_ of 40 μg/mL in minipigs, a single dose of subcutaneous gentamicin was administered at an allometrically scaled human equivalent dose of 7.5 mg/kg (8.25 mg/kg) and 11.25 mg/kg (12.375 mg/kg). The target C_max_ of 40 μg/mL was reached with 8.25 mg/kg. Five NF1 minipigs at 2 to 3 months of age were administered subcutaneous gentamicin once daily for 14 days alongside four untreated age-matched controls. Gentamicin was well tolerated with no significant changes in renal parameters, including serum creatinine and blood urea nitrogen. PK parameters reached a mean C_max_ of 38 μg/mL on day 1, and no significant changes in PK parameters were noted after 14 days, suggesting that gentamicin did not accumulate in the plasma over time (Figure 3A, Tables S3 and S4).

To determine the ability of gentamicin to penetrate the blood-brain and blood-nerve barriers and exert an effect in clinically relevant tissues, we performed PK and PD analyses on optic nerve, cortex, sciatic nerve, and skin from minipigs 30 min after gentamicin administration on day 14. Gentamicin was present in all tissues and reached a mean concentration of 5.87 ± 0.97 μg/mL in optic nerve, 1.01 ± 0.2 μg/mL in cortex, 6.88 ± 1.54 μg/mL in sciatic nerve, and 3.02 ± 1.28 μg/mL in skin (Figure 3B). Neurofibromin protein expression was higher in the optic nerve tissue of animals treated with gentamicin compared with untreated controls (p = 0.01), but no significant change was observed in other tissues (Figures 3C–3E). Neurofibromin protein was not detected in any skin sample and thus was not quantified. While p-ERK appeared to be downregulated after gentamicin treatment, this was not significant in any tissues (Figures 3F–3I).

We hypothesized that nonsense suppression treatment alone was not sufficient to cause a detectable change in neurofibromin protein expression in certain tissues and cell types. To determine the limit of detection for neurofibromin protein expression by western blot, we prepared protein lysates containing increasing percentages of total WT protein from primary Schwann cell cultures. Neurofibromin signal was detectable with as low as 2.5% WT protein, suggesting that nonsense suppression drugs increase neurofibromin protein expression by less than 2.5% in *NF1*^*NS/−*^ minipig Schwann cells (Figure S5). Ataluren has been shown to enhance aminoglycoside-induced readthrough by utilizing an orthologous mechanism of nonsense suppression.^25^ However, combination treatment with ataluren and gentamicin did not restore neurofibromin protein expression or reduce p-ERK in Schwann cells with LOH.

**Figure 4:**
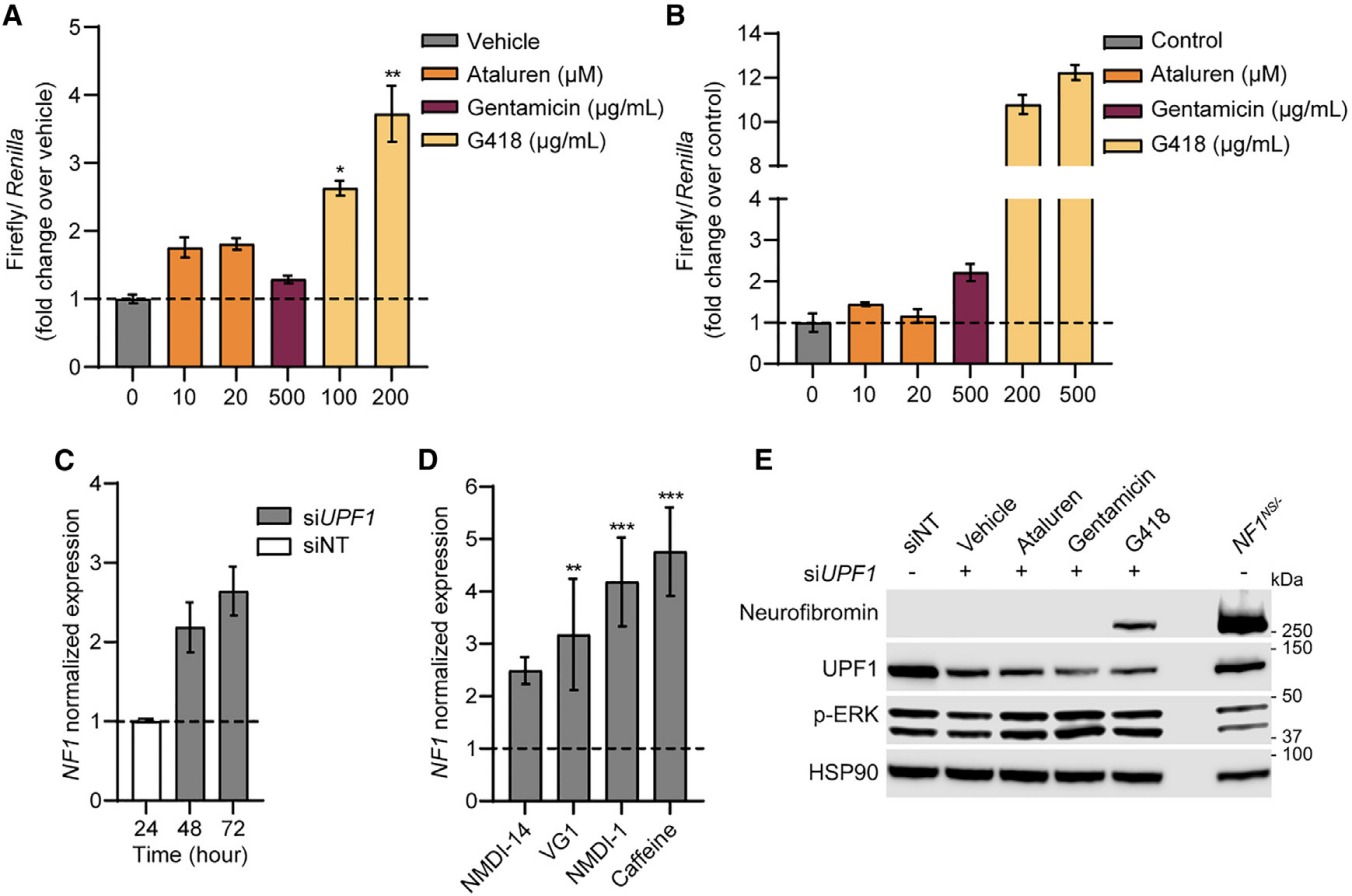
Pharmacologic and genetic inhibition of nonsense-mediated decay (NMDI) in *NF1*^*NS/−*^ cNF Schwann cells (A) Dual luciferase reporter assay showing a significant dose-dependent increase in firefly luciferase activity in *NF1*^*NS/−*^ cNF Schwann cells treated with G418 but not ataluren or gentamicin. Error bars represent mean and SD of three technical replicates. Unpaired t-test was performed (ns, not significant with p value > 0.05; ∗p value ≤ 0.05; ∗∗p value ≤0.01; ∗∗∗, p value ≤0.001; ∗∗∗∗, p value <0.0001). (B) Dual luciferase reporter assay showing a significant dose-dependent increase in firefly luciferase activity in *NF1*^*NS/−*^ Human Schwann cells (ipNF95.6) treated with G418 but not ataluren or gentamicin. (C) RT-qPCR analysis of *NF1* gene expression 48 and 72 h after siRNA-mediated knockdown of *UPF1* (si*UPF1*), normalized to non-targeting siRNA (siNT) after 24 h. (D) RT-qPCR analysis of *NF1* gene expression normalized to vehicle-treated 24 h after treatment with NMDI-14 (5 μM), VG1 (10 μM), NMDI-1 (5 μM), or 6 h after treatment with caffeine (7.5 mM). Error bars represent mean and SD of three technical replicates. Unpaired t-test was performed (ns, not significant with p value > 0.05; ∗p value ≤ 0.05; ∗∗p value ≤ 0.01; ∗∗∗p value ≤0.001; ∗∗∗∗p value < 0.0001). (E) Representative western blot showing increased neurofibromin protein expression with si*UPF1* in combination with G418 (100 μg/mL), but not ataluren (10 μM) or gentamicin (500 μg/mL).

Transgenic reporter systems are commonly used to measure ribosomal readthrough of nonsense mutations and have been used in high-throughput screens to identify new therapeutics.^26^ To explore this in the context of NF1, we stably transfected *NF1*^*NS/−*^ Schwann cells and human immortalized Schwann cells from an NF1 nonsense mutation-associated plexiform neurofibroma (iPNF95.6, ATCC CRL-3389) with a dual luciferase reporter (DLR) construct containing five codons of *NF1* sequence surrounding the *NF1*^*R1947∗*^ mutation.^27^ Previous studies have shown this to be sufficient to provide the local sequence context to reflect readthrough potential, and 33 nucleotides is about the same length as a ribosomal footprint, such that the ribosome would only be in contact with *NF1* mRNA sequence when bound to the PTC.^28^ This sequence is flanked by sequences encoding *Renilla* and Firefly luciferase. As a result, *Renilla* was expressed constitutively in transfected cells while firefly was only expressed if readthrough of the nonsense mutation was achieved. Treatment with G418, but not gentamicin or ataluren, caused a significant and dose-dependent increase in firefly luciferase activity compared with vehicle-treated cells in both minipig and human Schwann cells (Figures 4A and 4B). These results suggest that nonsense suppression of *NF1* may be possible in minipig and human Schwann cells but does not occur at a high enough rate to be detectable by traditional western blot or in the context of the full *NF1* gene.

As shown above, *NF1* transcript levels in *NF1*^*NS/+*^ Schwann cells represent about 50% of WT levels and are almost undetectable in Schwann cells with LOH (Figures 2C and S2A). This result suggests that NMD is strong for this allele. To further study the potential of NMDI to enhance nonsense suppression, we inhibited the NMD pathway by molecular targeting of *UPF1*, a critical mediator of the NMD pathway.^7^^,^^26^ *NF1*^*NS/−*^ Schwann cells were treated with siRNA targeting swine *UPF1*, which resulted in a 50% reduction in *UPF1* transcript corresponding to a 75% reduction in UPF1 protein expression (Figure S6), and a 2- to 3-fold increase in *NF1* mRNA expression compared with a non-targeting siRNA (Figure 4C). Pharmacologic NMDIs, including caffeine, NMDI-1, NMDI-14, and VG1 (an NMDI-1-like compound), have been shown to increase transcript levels and enhance nonsense suppression in other nonsense mutation models.^7^^,^^17^^,^^26^^,^^29^ Indeed, all NMDI compounds tested increased *NF1* mRNA expression in *NF1*^*NS/−*^ Schwann cells by 2- to 5-fold compared with vehicle, though this was not consistent between experiments (Figure 4D). To evaluate the ability of this combination to induce full-length endogenous neurofibromin protein, *NF1*^*NS/−*^ Schwann cells were treated with G418, gentamicin, or ataluren in combination with NMDI-14, VG1, or NMDI1 for 48 h. These combinations did not induce detectable neurofibromin protein expression in *NF1*^*NS/−*^ Schwann cells. However, treatment with *siUPF1* in combination with G418 restored detectable full-length neurofibromin protein expression in *NF1*^*NS/−*^ Schwann cells (Figure 4E). This result was attributable to nonsense suppression and not an alternative mechanism of increased protein expression because treatment of WT or *NF1*^*FS/−*^ Schwann cells with si*UPF1* and nonsense suppression drugs had no effect on neurofibromin protein expression (Figure S7). Interestingly, si*UPF1* did not enhance neurofibromin protein expression when combined with ataluren or gentamicin (Figure 4E).

## Discussion

In this study, we sought to evaluate nonsense suppression as a potential therapy for NF1. Nonsense suppression is an attractive candidate therapy for the 20% of NF1 patients with a heterozygous loss of function nonsense mutation,^1^ not only to treat NF1-associated tumors but also the other numerous manifestations of NF1 that are highly variable between patients and for which a small increase in full-length neurofibromin protein expression could alleviate some symptoms. However, the level of neurofibromin expression required to mitigate the effects of functional loss is unknown. This remains a critical gap in our knowledge, especially as it relates to tumor formation, tumor maintenance, and neurological function.

We examined three nonsense suppression therapies in our NF1 minipig model *in vitro*: gentamicin, ataluren, and G418. We were not able to detect readthrough of *NF1* by endogenous neurofibromin protein expression in primary Schwann cells from NF1 minipigs. We first tested these therapies in heterozygous Schwann cells with a nonsense mutation in one allele of *NF1*. It proved difficult to detect small changes in neurofibromin protein expression, which is attributable to a lack of sensitivity in western blotting for this large protein. When we did see increases in neurofibromin protein expression, they did not consistently correlate with p-ERK reduction. We therefore could not conclude that any reduction in p-ERK visualized in a particular experiment was a direct result of readthrough. It is possible that the difficulty in seeing consistent changes in p-ERK was due to the use of HSP90 as a housekeeping gene instead of ERK (which was chosen due to inadequate protein for multiple blots and difficulty multiplexing p-ERK and ERK), but this is unlikely since the changes were not consistent or based on any drug treatment that could have affected total ERK expression consistently. Schwann cells with LOH were a better candidate for screening, as it is easier to detect a small increase from 0% compared with a small increase from 50%. We found that only 2.5% of WT neurofibromin protein levels are required to detect neurofibromin protein by western blotting, but we could not detect this amount using these three traditional nonsense suppression therapies alone. Readthrough was evident by combining nonsense suppression with NMD inhibition, suggesting that NMD is an important barrier to nonsense suppression therapy (Figure 4E). Compared with other models that use immortalized cells with homozygous nonsense mutations or readthrough reporters, our model utilizes primary cells that have undergone spontaneous loss of the remaining WT allele. This more accurately represents the human disease, as neurofibromas develop due to spontaneous LOH in a subset of Schwann cells. However, we were unable to determine whether the loss of the WT allele was due to allelic conversion or a large deletion, and it is possible that the LOH allele in this model was not subject to nonsense suppression, thereby limiting the number of transcripts available for ribosomal readthrough. Additionally, the sequence context dependency of these therapies on the efficacy of readthrough induction is not well established, which may impact the efficiency of readthrough in our model.

Neurofibromin protein expression was successfully induced in primary *NF1*^*NS/−*^ Schwann cells by si*UPF1* in combination with G418, but not with ataluren or gentamicin. This is not surprising, as G418 is generally considered a stronger readthrough agent. Targeting NMD *in vivo* using siRNA is feasible, and the evaluation of si*UPF1* intratumorally in combination with nonsense suppression in the NF1 minipig is warranted based on our results. While the goal of these studies is to develop novel therapies for human patients with nonsense mutations in *NF1,* we were unable to induce neurofibromin protein expression using commercially available NMDIs in combination with G418, despite their ability to produce similar increases in *NF1* transcript when compared with si*UPF1* (Figures 4C and 4D). The pharmacologic NMDIs tested also affect UPF1 function and some have been shown to synergize with ribosomal readthrough in other preclinical models,^16^^,^^17^ so it was surprising that we were not able to induce readthrough with pharmacologic NMD inhibition in combination with G418. One possible explanation for this is that many of these models rely on transgenic readthrough reporters, which are more sensitive in detecting readthrough compared with endogenous protein expression readouts. Another explanation is that these combinations are sufficient to restore readthrough in other genes, but not *NF1*. Additionally, we had a small sample size of primary Schwann cell cultures with LOH, and future *in vitro* studies would be strengthened by evaluating multiple cell types of interest in NF1, including other cells that undergo LOH, like melanocytes associated with CALMs and oligodendrocytes associated with optic pathway glioma.

Based on promising early results showing a trend toward increased neurofibromin in primary *NF1*^*NS/+*^ Schwann cell cultures treated with gentamicin (Figures S3E and S3F), increased luciferase activity using a DLR (Figure 4A), and historical data supporting its use as a nonsense suppression therapy, we moved forward with evaluating the steady-state PK and PD of gentamicin *in vivo* in our minipig model of NF1. While this medication is not viable for long-term therapy in human patients, it represented a valuable proof-of-concept study, as it is commercially available and has been tested extensively in preclinical models and clinical trials.^5^^,^^6^^,^^7^^,^^20^^,^^22^^,^^23^^,^^24^ We were able to detect gentamicin in all tissues, although mean tissue gentamicin concentrations differed by up to 8-fold from cortex (lowest) to sciatic nerve (highest) (Figure 3B). We have previously shown that selumetinib, an MEK inhibitor recently approved for treatment of NF1-associated neurofibromas, reaches higher concentrations in the brain of NF1 minipigs compared with WT minipigs, which may be due to alterations in the blood-brain barrier.^10^ Gentamicin concentrations were lowest in the cerebral cortex, suggesting minimal brain penetrance despite the potentially leaky barrier. However, gentamicin concentrations were higher in the optic nerve and sciatic nerve compared with the skin. This suggests that gentamicin, for example, could reach therapeutic levels at a lower dose for NF1-associated optic pathway glioma compared with cutaneous manifestations of NF1, like neurofibromas. Indeed, gentamicin significantly increased full-length neurofibromin protein expression in NF1 minipig optic nerve at steady state (Figures 3B and 3C). Surprisingly, this did not occur in sciatic nerve, which reached similar gentamicin concentrations (Figures 3B and 3E). This discrepancy could be due to inherent differences in myelin structure and cellular composition between optic and sciatic nerve.^30^ We found that p-ERK was not significantly modulated in any tissue. The traditional way to detect neurofibromin loss of function is Ras hyperactivation, which can be detected using a Ras pulldown assay for active Ras, or ERK phosphorylation as a downstream marker of Ras activation. These measures are highly variable and often difficult to assess robustly. This variability is evidenced by our inconsistent results *in vitro*, where some individual cultures showed increased neurofibromin with paradoxically increased p-ERK (Figure S3B, 48H, 100 μg/mL). MAPK inhibition has been shown to cause negative feedback and can elicit a rebound effect, paradoxically increasing ERK phosphorylation over time.^31^^,^^32^ Transcript levels of other downstream transcription factor genes, including *DUSP6*, *ETV5*, and *FOS,* may be a more stable readout of alterations in MAPK signaling at steady state.^33^^,^^34^^,^^35^ Moreover, the amount of full-length neurofibromin protein expression required to reduce MAPK signaling and ameliorate clinical symptoms of NF1 is unknown and may differ between tissues and cell types. The results of this *in vivo* study suggest that tissue penetrance and readthrough efficacy of gentamicin is highly variable between tissues in NF1. This proved to be the case in NF1 minipig tissues after selumetinib administration and illustrates the value of the NF1 minipig as a model for testing tissue PK and PD of novel therapies prior to clinical trials, particularly with respect to the blood-brain and blood-nerve barriers.^10^

Using a PTC readthrough reporter of the *NF1*^*R1947∗*^ nonsense mutation, we showed that the three nonsense suppression drugs we were testing can induce readthrough in primary minipig Schwann cells and an immortalized human cell line, with G418 inducing more robust readthrough compared with other aminoglycosides and ataluren (Figures 4A and 4B). This is in line with other disease models using similar cell-based reporter systems. However, there are limitations to these systems. First, *NF1* is a large gene and only a short sequence surrounding the mutation was included in the construct. Thus, readthrough in the context of the full gene could not be evaluated using this measure. Second, the DLR construct does not contain introns, which is required for the NMD complex to exert its effect, so the impact of NMD on nonsense suppression could not be assessed, and we could not evaluate NMD inhibition using this assay. Last, the siRNA results have limitations, as only one of the three constructs (construct 1) gave consistent and prolonged UPF1 inhibition and was used for subsequent experiments (Figure S6). Increase in neurofibromin using siRNA treatment in combination with nonsense suppression therapy could not be replicated with siRNA constructs 2 or 3.

This is the first report of readthrough induction of full-length neurofibromin protein in a nonsense mutation model of NF1. In this study, we demonstrated that full-length neurofibromin protein is inducible in Schwann cells with a nonsense mutation and spontaneous LOH, but this required NMD inhibition and was a small percentage of WT levels. The lack of available therapies combining nonsense suppression and NMD inhibition is a major barrier to treating NF1 patients with nonsense mutations. The development of pharmacologic agents with minimal toxicity along with other methods including RNA therapy for NMD inhibition is needed. It will be important to determine how much neurofibromin protein expression will be sufficient to alleviate disease and develop new ways to augment readthrough of PTCs. In sum, the *NF1*^*R1947∗/+*^ minipig provides a valuable spontaneous model of NF1, which develops cutaneous neurofibromas and can be used to evaluate PK and PD of new readthrough agents in tissues of clinical significance. Primary *NF1*^*R1947∗/−*^ Schwann cells can be isolated from these neurofibromas and used effectively to assess new readthrough compounds using transgenic readthrough reporters and endogenous neurofibromin protein expression.

## Materials and methods

### Generation of NF1^NS/+^ minipigs

Generation of a swine nonsense mutation model of NF1 followed a similar strategy as previously described.^9^ Recurrent nonsense mutation *NF1*^*NS*^ is located within exon 41 of the swine *NF1* gene, which shares 100% amino acid homology with exon 39 in the human *NF1* gene (Ensembl accession number: ENSSSCG00000017748.4, ENSG00000196712.18). TALENs were designed to flank the region of *NF1*^*R1947*^ in exon 41. Ossabaw minipig fetal fibroblasts isolated from day 38 embryos were transfected with TALENs and a 90-mer homology-directed repair (HDR) template containing the *NF1*^*R1497∗*^ mutation, as well as three silent mutations to prevent TALEN rebinding. Transfection reactions included 0.6 × 10^6^ fibroblasts, 0.5 μg of RNA from each TALEN, and 0.2 nmol of HDR were pulsed for 20 ms at 1,800 V using the Neon Transfection System (Thermo). Resulting cells were plated at low density for 10 days at 38.5°C. Colonies derived from single cells were isolated, and gene editing was detected by DNA sequencing. Heterozygous clones were used to produce filial 0 (F0) generation *NF1*^*R1947/+*^ minipigs using somatic cell nuclear transfer. *NF1*^*R1947/+*^ F0 minipigs were sequence validated and bred with WT sows to produce offspring that exhibited germline transmission of the *NF1* allele mutation with Mendelian frequencies.

### Animal husbandry

All animal work was performed in Recombinetics facilities under its animal welfare assurance A4728-01. All animal protocols were reviewed and approved by the Institutional Animal Care and Use Committee and performed with appropriate veterinary oversight.

### Sequence validation

Validation of gene modification was performed after fibroblast transfection. Cells were lysed in PCR-safe lysis buffer (10 mM Tris·HCl, pH 8.0; 2 mM EDTA; 2.5% (vol/vol) Tween 20; 2.5% (vol/vol) Triton X-100; 100 μg/mL proteinase K) and incubated for 1 h at 55°C and 15 min at 95°C. PCR used 1 μL of prepared lysate in Accustart II PCR Supermix (QuantaBio) according to the manufacturer’s instructions. Gene editing in colonies was detected by direct Sanger sequencing of PCR amplicons. DNA was isolated from harvested tissues using DNeasy blood and tissue kit (Qiagen) according to the manufacturer’s protocol.

### Schwann cell culture

Schwann cells were isolated as previously described.^9^^,^^18^ Sciatic nerve or cutaneous neurofibroma were biopsied from WT or NF1^R1947/+^ minipigs and transported in Dulbecco’s Modified Eagle’s Medium (DMEM; Thermo) containing 10% fetal bovine serum (FBS; Thermo) and 1% Antibiotic/Antimycotic (AA; Thermo). In a laminar flow hood, specimens were rinsed in 70% ethanol and phosphate-buffered saline (PBS; VWR 16750-078) by briefly dipping into each solution 10 times. Tissues were minced and incubated at 37°C, 5% CO_2_ in dissociation media containing DMEM with 10% FBS, 1% AA, 1x collagenase A (Thermo), and 1x dispase II (Roche) overnight in a Petri dish, then transferred to a conical and centrifuged at 270 × *g* for 5 min. Cells were plated onto a poly-L-lysine (0.05 mg/mL) and laminin-coated (10 μg/mL) six-well plate and cultured in complete media containing DMEM with high-glucose (GIBCO) supplemented with 10% FBS (GIBCO), forskolin (2 μM) (Calbiochem), 1% N2 supplement (Thermo), recombinant human neuregulin-b1 (NRG1) (10 ng/mL) (R&D Systems), and 1% AA. Media was replaced every 2 days. Schwann cells were separated from contaminating fibroblasts via magnetic cell selection, as described.^9^^,^^18^ Purified Schwann cells were expanded and stored in liquid nitrogen at low passage and vials were thawed immediately prior to use in experiments to maintain low-passage cells.

### Flow cytometry

Schwann cells were isolated from contaminating fibroblasts via p75 NGFR antibody-mediated magnetic cell selection, according to the manufacturer’s protocol (MACS, Miltenyi Biotec).^7^ Positive (Schwann cells) and negative (fibroblasts) fractions were incubated with anti-p75 NGFR antibody (Table S5) for 15 min, then washed for 5 min with PBS +2% FBS. Unstained Schwann cells were used as a negative control after initial testing with an isotype control antibody showed no background staining. Cells were run through a CytoFLEX flow cytometer (Beckman Coulter) using the Cytexpert software according to the manufacturer’s protocol. FlowJo software was used to determine cell purity.

### Quantitative reverse transcription PCR

Total RNA was extracted from cells and tissues using the RNeasy mini kit (Qiagen) following manufacturer’s instructions. RNA concentration and quality was measured using a NanoDrop One (Thermo). RT-qPCR was performed using the Bio-Rad CFX96 Real-Time System (Bio-Rad) using technical replicates and Beta-actin as a housekeeping gene. Data were then normalized to the average of the control group and fold change was calculated as 2^−ΔΔCt^. Primers used for this study are listed in Table S6.

### RNA sequencing

RNA was extracted from cells using the RNeasy mini kit (Qiagen) following the manufacturer’s instructions. Samples were sequenced on an Illumina NovaSeq 6000 using a 150 paired end flow cell. For comparisons of transcript expression between fibroblasts and Schwann cells (Figures S2A and S2B), data were pooled from WT fibroblasts from skin (n = 1) and sciatic nerve (n = 1) to obtain n = 2, and data in the form of Fragmnets Per Kilobase of Transcript Per Million Mapped Reads (FPKM) were pooled from all Schwann cell cultures to obtain n = 15. For comparisons between Schwann cells, data were separated as follows: WT Schwann cells (n = 2), *NF1*^*NS/+*^ Schwann cells (n = 8), and *NF1*^*−/−*^ (pooled data from *NF1*^*NS/−*^ and *NF1*^*FS/−*^*)* Schwann cells (n = 5). The RNA sequencing data were mapped to the Ensembl Sus Scrofa genome 11.1, release 103^36^ using HISAT2^37^ and quantified with StringTie.^38^ All graphs were generated using R^39^ and tidyverse.^40^

### Protein extraction from cells and tissues

For extraction of protein from cells, snap frozen pellets were briefly thawed on ice and resuspended in RIPA buffer (Sigma) containing protease (Sigma) and phosphatase inhibitors (Sigma), then vortexed at 2,000 rpm at 4°C for 20 min, then centrifuged at 4°C for 15 min at 20,000 × *g*. For extraction of protein from tissues, snap frozen tissues were thawed briefly on ice, then 50 to 80 mg of tissue was transferred to a 2-mL microtube containing ceramic homogenizers (Agilent Technologies) and homogenized using a Bead Blaster (Benchmark Scientific) at 4,260 rpm, 7 m/s linear speed, 3 cycles of 30 s at 4°C. Homogenates were centrifuged at 13,000 rpm for 3 min at 4°C, then supernatants were transferred to new tubes and sonicated at an amplitude of 20A for 10 s at 4°C, vortexed at 2,000 rpm at 4°C for 20 min, then centrifuged at 20,000 × *g* at 4°C for 15 min.

### Western blotting

Protein lysates from cells or tissues were quantified using a BCA assay (Thermo). Twenty to 30 μg of protein was denatured at 95°C for 5 min and separated by electrophoresis using SDS on 1.5-mm NuPAGE 4–12% Bis-Tris protein gels (Thermo). Protein was transferred to Immun-Blot PVDF Membrane (Bio-Rad) overnight (16–20 h) at 18 V, 4°C. Membranes were cut according to target molecular weights and blocked at room temperature (RT) for 1 h using 5% blocking grade buffer (Bio-Rad) in TBST (Tris-buffered saline, 0.1% Tween 20) (Sigma). Membranes were incubated with primary antibody overnight at 4°C and washed with TBST three times for 5 min at RT, then incubated with horseradish peroxidase-conjugated secondary antibody for 1 h at RT and washed again as described above. All washes and incubations were performed on a rocking platform. Blots were developed with the WesternBright Quantum Kit (Advansta). All washes and incubations were performed on a rocking platform. Bands were imaged using the Li-Cor Odyssey Fc Imaging System, quantified by densitometry, and normalized to HSP90. Antibodies used for this study are listed in Table S5.

### Immunofluorescence

Schwann cells or fibroblasts were seeded on 8-well chamber slides (Ibidi) coated with poly-L-lysine and laminin, as described.^9^ Cells were harvested 24 to 48 h later and fixed in 4% paraformaldehyde (EM Sciences) for 15 min at RT. Fixed cells were permeabilized in PBS containing 1% bovine serum albumin and 0.2% Triton X-100 for 30 min at RT, and further incubated with anti-p75 NGFR overnight at 4°C in the dark. The next day, cells were washed in PBS containing 1% BSA two times for 5 min at RT, incubated with anti-phalloidin for 1 h in the dark at RT, and washed again as described above. Cells were mounted with ProLong Gold Antifade Mountant with DAPI (Thermo) and coverslipped using Gold Seal Cover Glass (EM Sciences). Images were acquired using a Cytation 5 cell imaging reader (BioTek).

### Proliferation assay

Schwann cells were seeded in triplicate at 1,200 cells per well in poly-L-lysine and laminin-coated 96-well plates and allowed to adhere overnight in complete media. Media was added to empty wells as a background control. Proliferation was evaluated by the alamarBlue^TM^ cell viability assay (Invitrogen) after indicated days in culture. Reagent was added to cells for 3 h, then fluorescence was detected using a Cytation 5 microplate reader (BioTek) according to the manufacturer’s protocol.

### Nonsense mutation suppression treatment of Schwann cells

Gentamicin (Sigma) and G418 (Corning) were reconstituted in sterile deionized water to a stock concentration of 50 mg/mL and 500 mg/mL, respectively, and stored at −20°C. Ataluren (MedChem Express) was reconstituted in sterile dimethyl sulfoxide (DMSO) to a stock concentration of 10 mM and stored at −80°C. Cells were seeded in complete media at a density of 0.3 × 10^6^ cells/plate in 10-cm plates. Media was changed 24 h later to complete media without AA and containing vehicle or drugs at indicated concentrations. Cells were harvested 24, 48, and 72 h after drug treatment and divided for protein and/or RNA extraction.

### *In vivo* drug formulation and dosing

Gentamicin was purchased from Fresenius Kabi LLC and formulated in sterile isotonic saline to a concentration of 40 mg/mL. Human equivalent doses (HED) were determined by allometric scaling using the standard conversion coefficient (K_m_), where K_m_ = 1.1 for minipig and HED determination. Consequently, a 7.5-mg/kg human equivalent dose was 8.25 mg/kg. The required volume of drug for each animal was then based on individual body weight and determined on the day of administration.

### Trial design

The PK of gentamicin in plasma was assessed at baseline and at a steady state, and the PD of gentamicin was assessed by measuring neurofibromin and p-ERK protein levels at steady state. For baseline evaluation, five NF1 minipigs at 2 to 3 months of age received a single subcutaneous injection of 8.25 mg/kg gentamicin. Whole blood was collected immediately before drug administration and at 0.5, 1, 2, 4, 6, 8, and 24 h after gentamicin administration. For steady-state PK analysis, five NF1 pigs received a subcutaneous injection of 8.25 mg/kg gentamicin daily for 14 days alongside four untreated age-matched controls. On day 14, blood was collected immediately prior to gentamicin administration and 0.5, 1, 2, 4, 6, 8, and 24 h after gentamicin administration. For tissue analyses, a final dose of gentamicin was administered on day 15 and animals were euthanized 30 min after administration. Tissue samples (skin, sciatic nerve, optic nerve, and cortex) were collected from each animal at the same approximate locations and divided for analysis.

### Quantification of gentamicin in plasma and tissues

Plasma isolations were performed as described in a previous study.^10^ Briefly, serial whole blood samples (1.5 mL) were collected from the jugular vein into lithium heparin vacutainer tubes. Samples were immediately transferred to a 1.7-mL microfuge tube and centrifuged at 2,000 × *g* for 10 min at 4°C. Supernatant (plasma) was transferred to a cryovial and stored at −80°C until analysis. For plasma specimens, gentamicin was measured with a standard immunoassay (Siemens Dimension Vista System and Flex reagent cartridge). Assay analytical measurement ranged from 0.2 to 12 mg/L, and samples with results higher than 12 mg/L were diluted appropriately and re-analyzed. Within-run precision is 0.3% CV, between-day precision is 0.4% CV, and accuracy is 99.8% (correlation coefficient). For each tissue type (skin, sciatic nerve, optic nerve, and cerebral cortex), samples were analyzed commercially using liquid chromatography with tandem mass spectrometry (LC-MS/MS; BioAgilytix, San Diego, CA). Gentamicin sulfate salt (Toronto Research Chemicals, Inc.) was used to prepare stock standard and working standard solutions. Gentamicin-d_35_ in its propyl derivative form (Sigma Aldrich, derivatized at BioAgilytix) was prepared as an internal standard. Standard curves and quality controls were prepared with untreated tissues. Tissues were prepared with a homogenization solution containing collagenase. Homogenates were mixed with internal standard (Gentamicin-d35), and derivatized with propyl chloroformate. The analysis of gentamicin isoforms C1, C1_a_, and C2 + C2_a_ (combined) was conducted via LC-MS/MS. The LC-MS/MS system consisted of a Waters Acquity UPLC (Milford, MA) coupled with a Waters Xevo TQ-S Micro Triple Quad mass spectrometer (Milford, MA). Mass transition data were acquired in electrospray positive ion (ESI^+^) mode and captured using MassLynx 4.1 (Waters, Milford, MA). The method is applicable for measuring total gentamicin concentrations ranging from 2.00 to 5,000 ng/mL. The mean percent deviations of quality control samples from the theoretical values did not exceed 5.46% from theoretical values.

### Plasma PK analysis

Gentamicin plasma concentration-time data from subcutaneous administration were analyzed with noncompartmental methods as implemented in R (version 4.0.1) and R Studio PKNCA package (version 0.9.4). Pharmacokinetic parameters and measures of systemic exposure included area under the concentration-time curve (AUC; linear up, log down), maximum concentration (C_max_), time to C_max_ (T_max_), and half-life (t_1/2_).

### DLR assay

The *NF1*^*R1497X*^ DLR plasmid was kindly provided by David Bedwell at University of Alabama. The *Renilla*-NF1-R1947X-Firefly insert contains 30 nucleotides surrounding the premature termination codon in NF1-R1947X inserted between the *Renilla* and Firefly genes. The 30-base pair readthrough cassette contains *NF1*^*R1497X*^ (G TCG ACG GAT GCC AAA TGA CAA AGA GTT GGA TCC). Plasmids were amplified in DH5a *E. coli* using ampicillin selection and purified using a Qiagen miniprep kit, according to the manufacturer’s protocol. To produce stable cell cultures, we used Gateway cloning to transfer the DLR insert to a hygromycin-resistant PiggyBac Sleeping Beauty (PBSB) transposon vector. Briefly, a GBlock containing the DLR insert sequence and attB sites was cloned into the pDONR221 vector using BP clonase to produce pENTR-Renilla-NF1-R1947X-Firefly. This was cloned into the PBSB vector using LR clonase to produce pPBSB-CG-Renilla-NF1-R1947X-Firefly-PGK-Hygro, which was confirmed by DNA sequencing. Schwann cells were grown to 80% confluency and transfected with the PBSB transposon and PB7 (PiggyBac transposase) (5 μg each/ 1million cells) using the Neon electroporation system (Invitrogen #MPK10096). Media was changed after 24 h, and cells were allowed to recover in complete media for 24 h. Hygromycin was added to the media for 5 days and stable transfectants were confirmed by *Renilla* luciferase activity. Cells were treated with nonsense suppression drugs for 48 h. The DLR assay system (Promega) was used to detect readthrough, according to the manufacturer’s instructions. Luminescence was measured using the Cytation 5 plate reader. Firefly luciferase activity was normalized to *Renilla* luciferase activity and reported as a fold change over vehicle-treated cells.

### siRNA transfection

Schwann cells were seeded at 0.15 × 10^6^ cells in 10-cm plates. Twenty-four hours later, cells were transfected with 50 nm of siRNA targeting *UPF1*. In a pilot experiment, three custom and non-overlapping UPF1 duplexes were tested (Figure S6):

(1) Sense: 5′-CUGUAAUGGACGUGGAAAUUU-3′

Antisense: 5′-AUUUCCACGUCCAUUACAGUU-3'

(2) Sense: 5′-GCAACAUAGCUGUGGAUCAUU-3′

Antisense: 5′-UGAUCCACAGCUAUGUUGCUU-3′

(3) Custom UPF1 duplex, 3 of 3: Sense: 5′-GGGUCAUUAUUGUGGGCAAUU-3′

Antisense: 5′-UUGCCCACAAUAAUGACCCUU-3′

Construct number 1 was chosen for further experiments, as it was the only construct that gave consistent and prolonged UPF1 inhibition. Increase in neurofibromin using siRNA treatment in combination with nonsense suppression therapy could not be replicated using siRNA constructs 2 or 3. Media was changed to fresh complete media 24 h later, and cells were harvested for protein at 48 h, 72 h, and 96 h post-transfection. Positive control Lamin A/C Control siRNA (Human/Mouse/Rat) (siGENOME #D-001050-01) used for the pilot experiment did not result in knockdown of minipig Lamin A/C, suggesting lack of sequence specificity with minipig and was therefore not used in subsequent experiments. Subsequent experiments utilized custom UPF1 duplex 1 of 3. Non-targeting siRNA (siGENOME #D-001210-01) and DharmaFECT 1 reagent (Horizon Discovery #T-2001-03) were used for all experiments according to the manufacturer’s protocol. For dual siUPF1 knockdown/nonsense suppression experiments, on day 3 post-transfection, cells were treated with vehicle or nonsense suppression drugs for 24 or 48 h, then harvested for RNA and protein extraction.

### Statistical analysis

All graphs and statistical analyses were performed using GraphPad Prism V9 with the exception of RNA sequencing analysis, which was performed using R. Specific analyses were used as indicated in figure legends to generate p values with α = 0.05 (ns, not significant with p value >0.05; ∗p value ≤ 0.05; ∗∗p value ≤ 0.01; ∗∗∗p value ≤ 0.001; ∗∗∗∗p value < 0.0001).

## Data Availability

The data that support the findings of this study are available from the corresponding author upon reasonable request.

## Supplemental information

Document S1. Figures S1–S7 and Tables S1–S6

Document S2. Article plus supplemental information
