# Supplementary material for: Combining nonsense mutation suppression therapy with nonsense-mediated decay inhibition in neurofibromatosis type 1
Source: Mol Ther Nucleic Acids. 2023 Jun 26;33:227–39. doi: 10.1016/j.omtn.2023.06.018 (PMC10384610; doi:10.1016/j.omtn.2023.06.018)
Supplement: Document S1. Figures S1–S7 and Tables S1–S6 [file mmc1.pdf]

OMTN, Volume *33*

Supplemental information

Combining nonsense mutation suppression therapy with nonsense-mediated decay inhibition in neurofibromatosis type 1

Sara H. Osum, Eunice I. Oribamise, Stanislas M.A.S. Corbière, Mandy Taisto, Tyler Jubenville, Alex Coutts, Mark N. Kirstein, James Fisher, Christopher Moertel, Ming Du, David Bedwell, David A. Largaespada, and Adrienne L. Watson

Table S1: LOH penetrance in Schwann cells derived from *NF1*$^{NS/+}$ minipig cutaneous neurofibroma.

Animal ID	Tumor ID	LOH?
**3266**	1	Yes
**3266**	2	No
**2683**	1	No
**2683**	2	No
**2683**	3	No
**3451**	1	No

Table S2: In vitro readthrough drug concentrations.

Drug	Dose 1	Dose 2	Dose 3
**Ataluren**	0 μM	10 μM	20 μM
**Gentamicin**	0 μg/mL	250 μg/mL	500 μg/mL
**G418**	0 μg/mL	100 μg/mL	200 μg/mL

Table S3: Day 0 plasma pharmacokinetic analysis of gentamicin in NF1 minipigs.

	Animal ID	Cmax (mg/L)	AUC 8 (hour*ng/mL)	AUC inf (hour*ng/mL)	Kel	$t_{1/2}$ (hour)	Volume (L)	CL (L/hr)
	3800	32.4	53.4	55.7	0.382	1.82	8.11	3.1
	3802	30.7	54.4	55.8	0.443	1.56	7.59	3.36
	3814	35.2	73.2	74.9	0.468	1.48	4.92	2.3
	3815	38.3	70	73.6	0.336	2.06	7	2.34
	3831	53.4	65.7	68	0.462	1.5	5.01	2.32
**Mean**		38	63.34	65.6	0.4182	1.684	6.53	2.68
**Standard Deviation**		9.08	9.03	9.36	0.06	0.25	1.48	0.51

Table S4: Day 14 plasma pharmacokinetic analysis of gentamicin in NF1 minipigs.

	Animal ID	Cmax (mg/L)	AUC 8 (hour*ng/mL)	AUC inf (hour*ng/mL)	Kel	$t_{1/2}$ (hour)	Volume (L)	CL (L/hr)
	3800	49.1	63.8	65.9	0.376	1.84	8.77	3.3
	3802	45.9	62.8	63.9	0.456	1.52	7.72	3.52
	3814	42.5	70.3	72.8	0.356	1.95	7.8	2.78
	3815	41	71.1	73.8	0.359	1.93	6.95	2.34
	3831	35.2	62.8	64.2	0.434	1.6	7.14	3.09
**Mean**		42.74	66.16	68.12	0.3962	1.768	7.676	3.006
**Standard Deviation**		5.25	4.17	4.80	0.05	0.20	0.71	0.46

Table S5: Antibodies.

Antibody	Source	Dilution
Neurofibromin	CST 14623	1:1000
HSP90	CST 4874	1:3000
p-ERK	CST 4370	1:1000
UPF1	CST 9435	1:1000
Vinculin	CST 13901	1:3000
Anti-mouse IgG-HRP	CST 7076	1:10,000
Anti-rabbit IgG-HRP	CST 7074	1:5000
CD271 (p75 NGFR) PE	Invitrogen 12-9400-42	1:200
Phalloidin Alexa Fluor™ 488	Thermo A12379	1:200

Table S6: Primer sequences.

Target	Sequence
NF1	GCAGTTCAGACCCTAGTTTAC
	TGTTGGCTGGGATACATAACC
b-Actin	CGCTACCAGTTCGCCATG
	AAAGCCGGCCTTGCACAT
UPF1	ATATGCCTGCGGTACAAAGG
	ACACTGCTCCGAAGCTCAAT

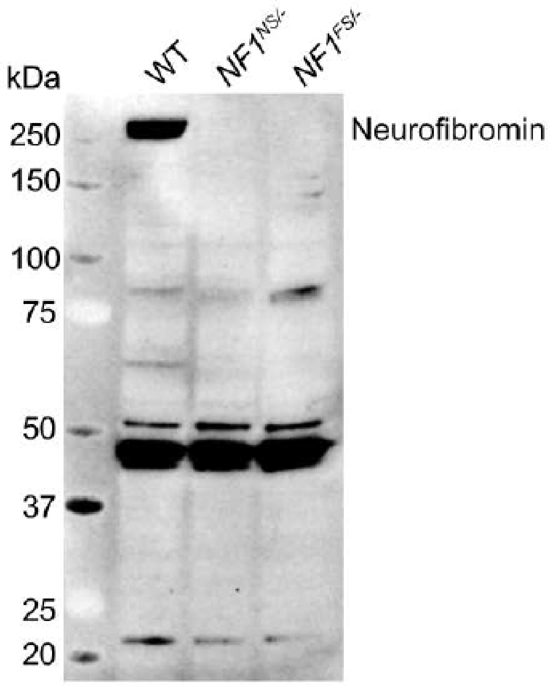

Figure S1: Full length Western blot showing no truncated neurofibromin protein expression in *NF1*$^{NS/-}$ and *NF1*$^{FS/-}$ Schwann cells. Strong band ~ 50kDa is attributable to nonspecific binding to the porcine immunoglobulin heavy chain.

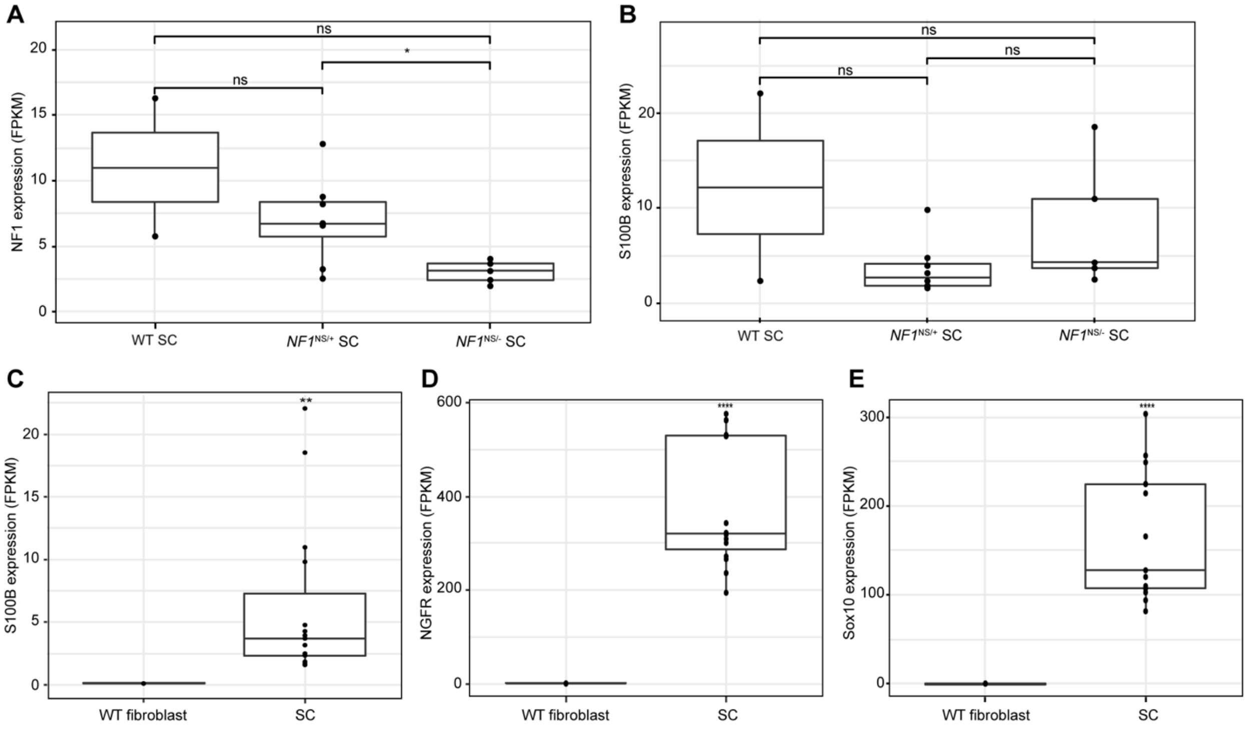

Figure S2: RNA sequencing analysis of *NF1* (A) and *S100B* (B) expression (FPKM) in WT Schwann cells (n=2), *NF1*$^{NS/+}$ Schwann cells (n=8), and *NF1*$^{-/-}$ (*NF1*$^{NS/-}$ and *NF1*$^{FS/-}$) Schwann cells (n=5). *S100B* (C), *NGFR* (D), and *SOX10* expression (FPKM) in WT fibroblasts (n=2) compared to SCs (pooled data from WT, *NF1*$^{NS/+}$, *NF1*$^{NS/-}$ and *NF1*$^{FS/-}$ Schwann cells). Error bars represent mean and SEM. Unpaired t-test was performed in R (ns, not significant with p-value >0.05; *, p-value ≤0.05; **, p-value ≤0.01; ***, p-value ≤0.001; ****, p-value <0.0001).ns, not significant with p-value >0.05; *, p-value ≤0.05; **, p-value≤0.01; ***, p-value≤0.001; ****, p-value<0.0001).

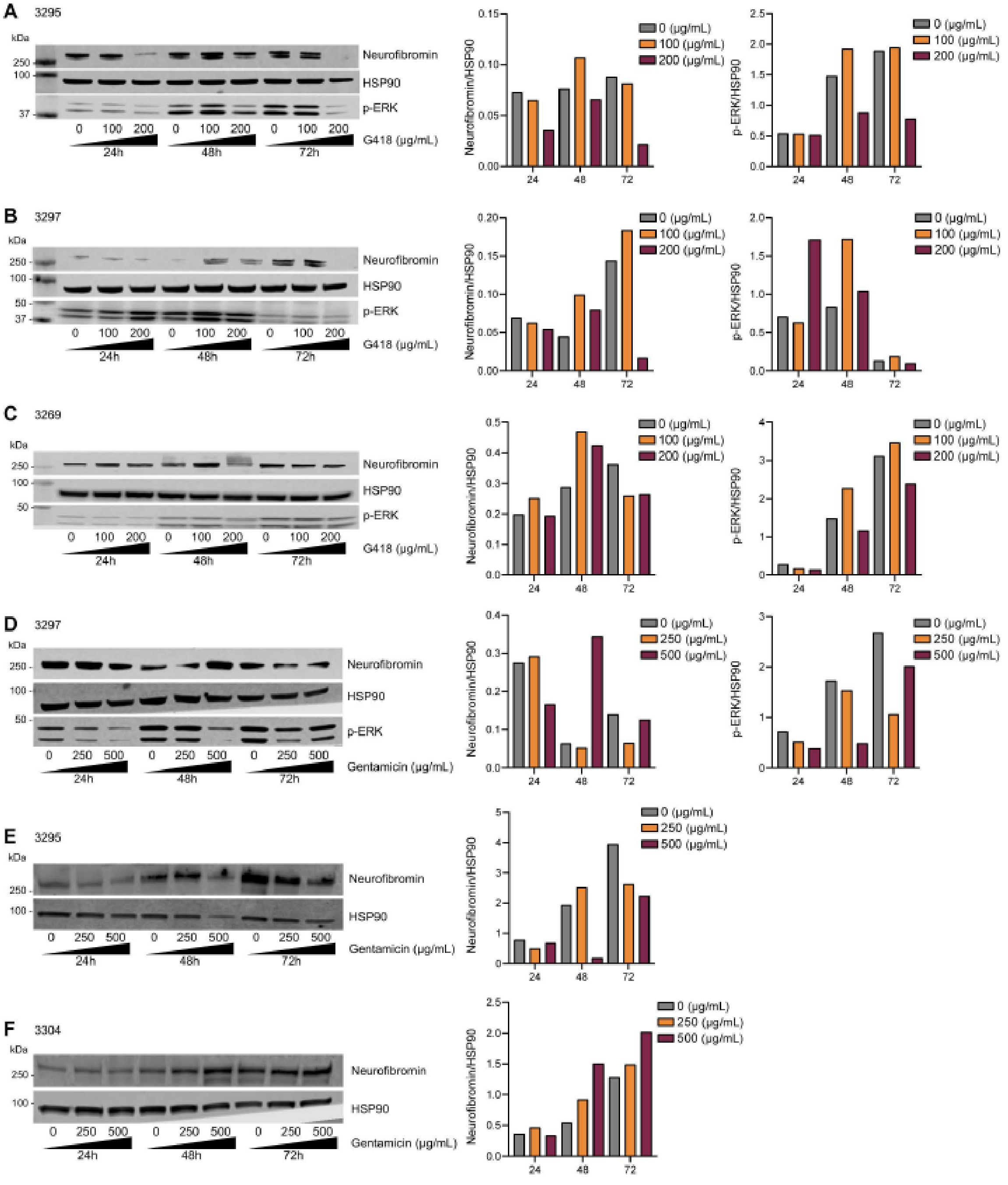
A 3295
B 3297
C 3269
D 3297
E 3295
F 3304
kDa
Neurofibromin
HSP90
p-ERK
G418 (µg/mL)
Gentamicin (µg/mL)
0 100 200
0 250 500
24h
48h
72h
Neurofibromin/HSP90
p-ERK/HSP90
0 (µg/mL)
100 (µg/mL)
200 (µg/mL)
250 (µg/mL)
500 (µg/mL)
24
48
72

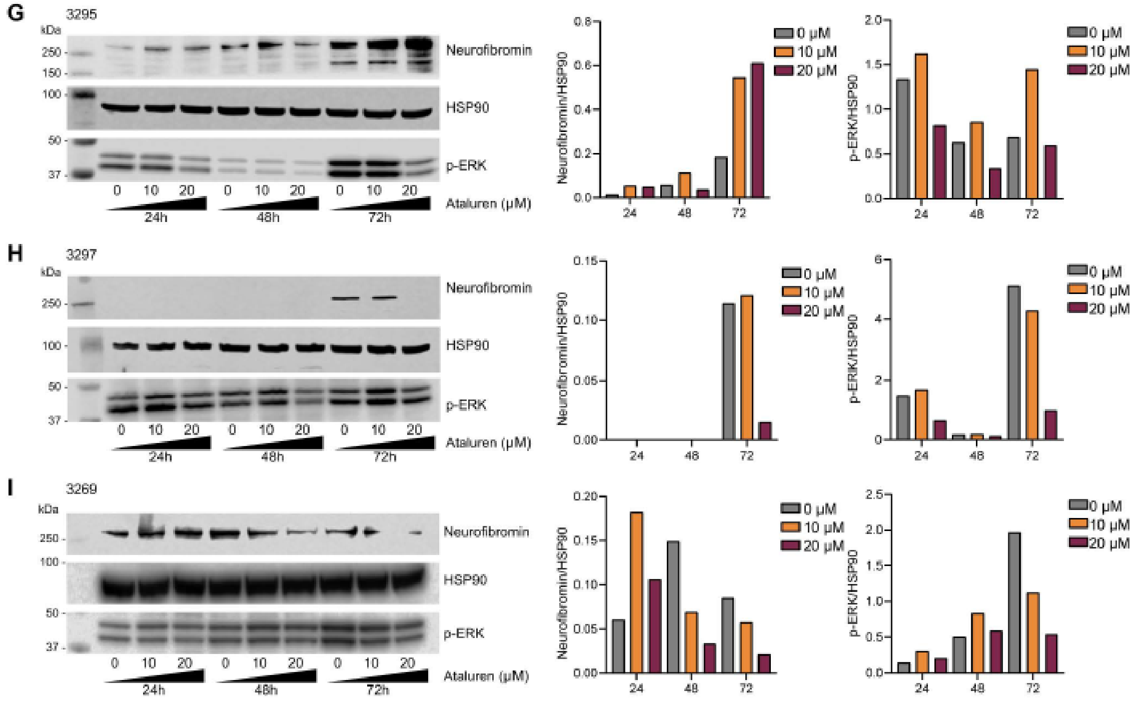

Figure S3: Western blots of neurofibromin protein expression in *NF1*$^{NS/+}$ Schwann cells (n=3 biological replicates) after treatment for 24, 48 or 72 hours with 0 (vehicle), 100, or 200μg/mL G418 **(A-C)**, 0 (vehicle), 250 or 500 μg/mL gentamicin **(D-F)**, or 0 (vehicle), 10, or 20 μM ataluren **(G-I)**.

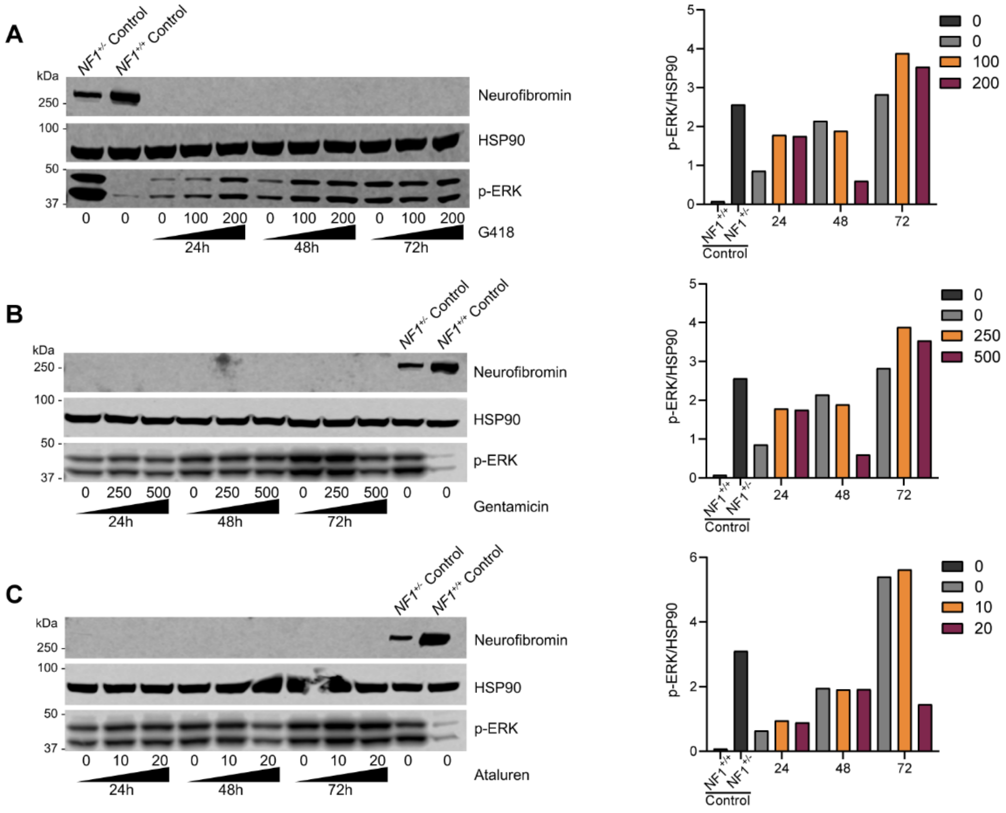

Figure S4: Neurofibromin protein expression in *NF1*$^{NS/-}$ Schwann cells after treatment for 24, 48 or 72 hours with 0 (vehicle), 100, or 200µg/mL G418 **(A)**, 0 (vehicle), 250 or 500 µg/mL gentamicin **(B)**, or 0 (vehicle), 10, or 20 µM ataluren **(C)**. Heat Shock Protein 90 (HSP90) is used as a loading control and housekeeping gene for both target proteins Neurofibromin (nonsense suppression target) and p-ERK (MAPK readout).

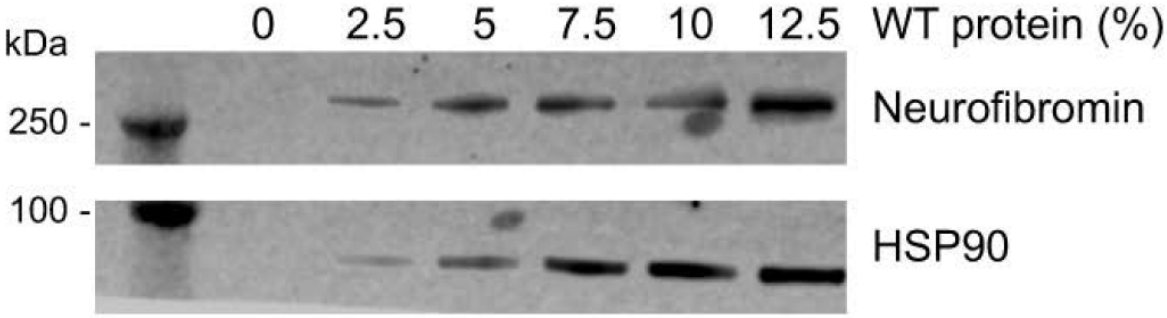

Figure S5: Western blot of neurofibromin protein expression with increasing percentage of WT protein lysate (0-12.5%). Heat Shock Protein 90 (HSP90) is used as a loading control.

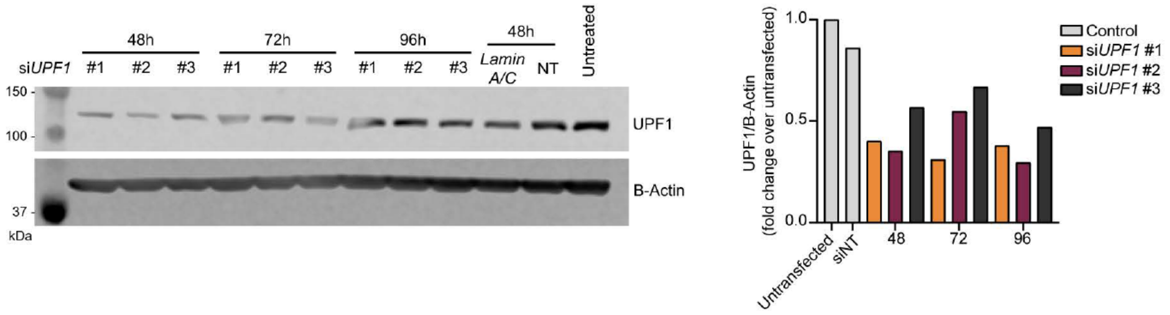

Figure S6: siUPF1 pilot experiment. *NF1*$^{NS/-}$ Schwann cells were treated with 3 different custom small interfering RNA (siRNA) oligos targeting swine *UPF1* (*siUPF1* #1, *siUPF1* #2, *siUPF1* #3), positive control siRNA targeting Lamin A/C (*Lamin A/C*), or negative control non-targeting siRNA (NT) and harvested 48h, 72h, or 96h post-transfection. Left panel – Western blot of UPF1, Lamin A/C and beta-actin (B-Actin, loading control) protein expression. *Lamin A/C*-targeting siRNA did not result in reduction of Lamin A/C protein, which is attributed to it being not cross-reactive with minipig *Lamin A/C*. Right Panel – Densitometry analysis of UPF1/B-Actin Western blot showing up to 75% knockdown of UPF1 sustained for 72h post-transfection with *siUPF1* #1.

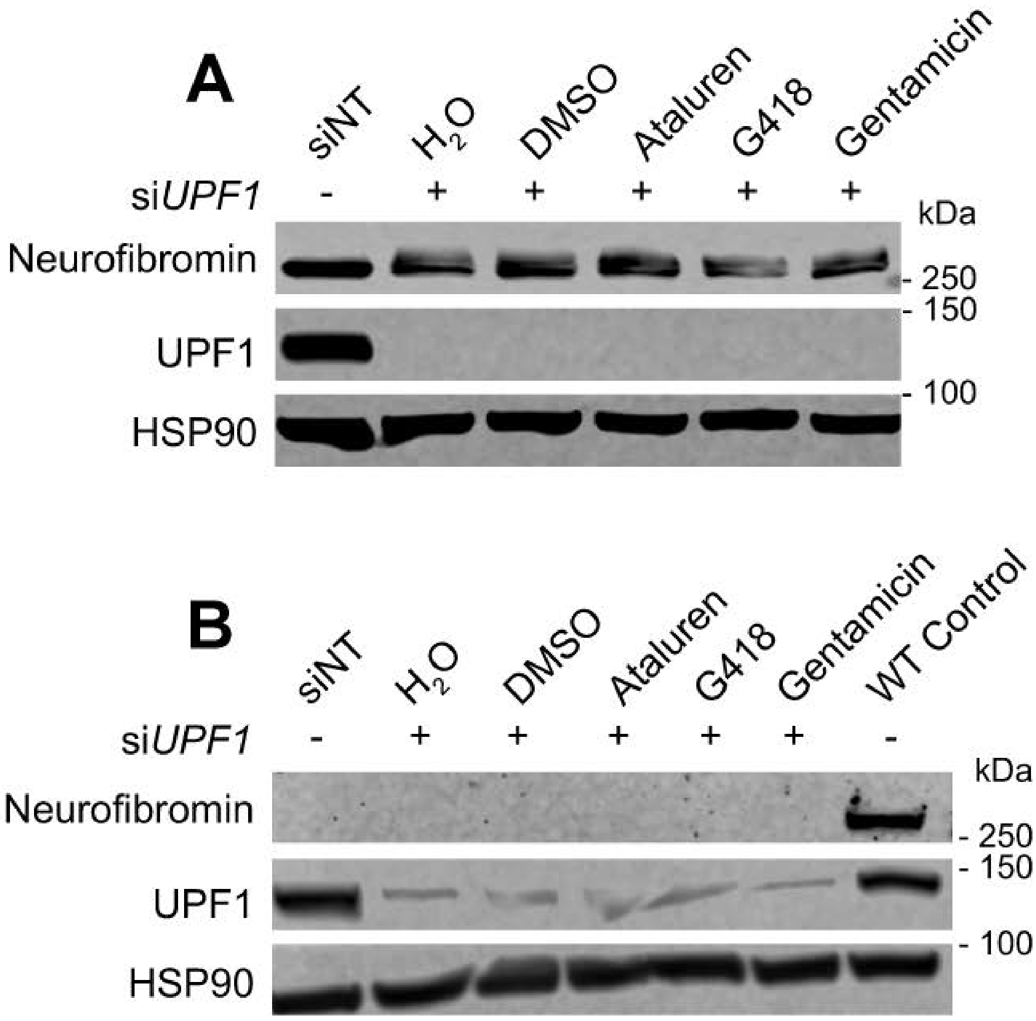

Figure S7: Western blot of neurofibromin and UPF1 protein expression after si*UPF1* knockdown in **(A)** WT and **(B)** *NF1*$^{FS/-}$ Schwann cells.
